# Experimentally induced puromycine aminonucleoside nephrosis (PAN) in rats: evaluation of angiogenic protein platelet-derived endothelial cell growth factor (PD-ECGF) expression in glomeruli

**DOI:** 10.1186/1423-0127-19-24

**Published:** 2012-02-16

**Authors:** Ismail Seckin, Mumin Uzunalan, Meltem Pekpak, Sibel Kokturk, Huseyin Sonmez, Zeynep Öztürk, Sibel Demirci, Elif Yaprak

**Affiliations:** 1 Department of Histology and Embryology, Cerrahpasa Faculty of Medicine, Istanbul University, Istanbul, Turkey; 2 Department of Nephrology, Cerrahpasa Faculty of Medicine, Istanbul University, Istanbul, Turkey; 3 Department of Histology and Embryology, Faculty of Medicine, Kocaeli University, Kocaeli, Turkey; 4 Department of Biochemistry, Cerrahpasa Faculty of Medicine, Istanbul University, Istanbul, Turkey

**Keywords:** puromycine aminonucleoside nephritis, PD-ECGF, angiogenesis, macrophage, ultrastructure, rat

## Abstract

**Background:**

In experimentally induced puromycine aminonucleoside nephrosis (PAN) animal models, nephrotic syndrome with minimal change disease and focal and segmental sclerosis-like nephritis similar to that in human is demonstrated; however, the real mechanism of PAN is not yet elucidated. Platelet derived endothelial cell growth factor (PD-ECGF), an endothelial mitogen protein, is believed to take part in microvessel formation and in stimulation of angiogenesis and its expression has not been totally demonstrated in PAN rats yet. In this study, we aimed to examine PD-ECGF expression in acute and chronic PAN induced in rats and find out the association between its expression and the stages of angiogenesis in kidney.

**Methods:**

For the experiment, twenty-four Male Wistar Albino rats were used and divided into four groups; control group (n = 6), pre-proteinuria group (n = 6), acute group (n = 6) and chronic group (n = 6). We compared statistically all data by One-way ANOVA Test followed by Dunn Multiple Comparison Test.

**Results:**

Proteinurea levels in control and pre-proteinuria groups were not statistically different; however, it was remarkably higher in the acute nephrosis group and significantly greater in the chronic nephrosis group than control group (*p *< 0.0025). In pre-proteinuria group, the serum albumin and creatinine clearances also did not significantly differ from the control group. On the other hand, in the acute and chronic nephrosis groups, serum albumin and creatinine clearances progressively decreased (*p *< 0.05). In our immunohistochemical studies, we showed elevated PD-ECGF expression in glomeruli of acute and chronic PAN rats. Microscopic and ultrastructural appearances of the glomeruli of acute and chronic PAN showed various sequential steps of angiogenesis, macrophages and immature capillaries with primitive lumens and apoptotic endothelial cells in the increased mesangial matrix.

**Conclusions:**

It is reported that acute and chronic PAN progressively increase PD-ECGF expression and following induction of angiogenesis in the affected glomeruli.

## Background

Experimentally induced puromycine aminonucleoside nephrosis (PAN), generally used as a model for podocyte injury, has the increase of mesangial matrix in glomeruli leading to massive proteinuria. It has been similar to the effects of minimal change disease and focal and segmental sclerosis-like nephritis in human [1-7]. However, the real mechanism of PAN is not yet elucidated.

Platelet derived endothelial cell growth factor (PD-ECGF/thymidine phosphorylase), isolated as an endothelial mitogen from platelets, is a 45-kDa angiogenic protein which stimulates the growth and chemotaxis of endothelial cells in vitro and angiogenesis in vivo [8-16]. Various studies have shown that PD-ECGF is one of the potent promoters of angiogenesis and mediates angiogenesis during many physiological and pathophysiological processes. Main sources of PD-ECGF are the infiltrating cells and especially macrophages, however, the mechanisms by which PD-ECGF contributes to angiogenesis are still unclear [8,10,17-21]. PD-ECGF is also expressed in the endothelium of various tissues [22].

It was reported that PD-ECGF expression is elevated in areas of interstitial fibrosis in scarred kidneys because local oxygen supply is most likely to be diminished in these areas due to obliteration of the postglomerular capillary network, tubules and fibroblasts and that its level of expression is correlated with the number of microvessels in various pathological conditions [17,20,22-24]. Moreover, experimental and clinicopathological studies have shown that the losses of podocytes [25] and renal capillaries [26] causing reduction of oxygen and nutritional supply to the kidney are closely linked with chronic disease progression and renal scarring. As known, hypoxia is a common stimulus for both angiogenesis and inflammation leading to the accumulation of macrophages and other immune cells [27], and for increased production of growth factors [28-31].

In this study, it was aimed to investigate PD-ECGF expression and angiogenesis in the glomeruli of acute and chronic puromycine aminonucleoside (PA) induced nephrotic rats with the view of the fact that hypoxia which might be developed due to loss of existing capillaries within the mesangial matrix may cause angiogenesis.

## Methods

### Preparation of Animals

Twenty-four young male Wistar albino rats weighing 90-120 g (Experimental Animals Reproduction and Research Laboratory, Istanbul University Cerrahpasa Medical Faculty, Turkey) were housed in individual cages in a temperature- and humidity- controlled room with a 12-h light/dark cycle. They were fed with standard rat chow and had free access to tap water.

Rats were divided into four groups with one control and three experimental groups (n = 6). The injection amounts, intervals and sacrification days are given in Table 1. Group I served as control and begining from the second day, control group was daily injected by 1 ml isotonic sodium chloride. Control rats were sacrificed at 10^th ^day. Three experimental groups were daily injected by 1.67 mg puromycine aminonucleoside (PA) (*Sigma *Chemical Co St. Louis, MO, USA) per 100 g body weight in 1 ml isotonic sodium chloride subcutaneously. Proteinuria developed in all PA injected rats at 6th day after the 5th injection. According to these parameters, we constituted two experimental groups. Group II, the "pre-proteinuria group", was killed on day 4 after 3th injection. Group III, the "acute nephrosis group", was sacrified on day 10 after 9th injection. The group IV, "chronic group", was created as described by Marinides (1990). Group IV rats received the same dose of PA once a week in the first three weeks, once in two weeks in the following weeks subcutaneously and they were killed after the 7th injection in 12 weeks (See Table 1). All rats were sacrified under ether anesthesia at the end of the study according to the regulation of animal ethical committee of Istanbul University.

**Table 1:** Injection intervals and amounts of all groups

Groups	Injection Intervals	Injection Amount	Total Injection Number	Sacrification day
Group I: Control	Daily	1 ml isotonic NaCl	9	10^th ^day

Group II: Pre-proteinurea	Daily	1.67 mg PA per 100 g body weight in 1 ml isotonic NaCl	3	4^th ^day

Group III: Acute Nephrosis	Daily	1.67 mg PA per 100 g body weight in 1 ml isotonic NaCl	9	10^th ^day

Group IV: Chronic	Weekly in first 3 weeks Biweekly in the rest weeks	1.67 mg PA per 100 g body weight in 1 ml isotonic NaCl	7	12^th ^week

The urine sample of each rat was daily collected and protein level was measured using the modified trichloracetyl acid (TCA) method [32]. All animals were weighed and their blood samples were taken for measurement of serum albumin and creatinine values (Jaffe) [20] (Table 2). Serum albumin levels were measured in clinical chemistry analyzer (Architect C8000, Abbott, Illinois, USA) using reagents purchased by the same manufacturer.

**Table 2:** All groups: Proteinuria, Serum Albumin, Creatinine -Clearance, Weight

Groups		Proteinuria mg/24 hours	Serum Albumin g/dl	Creatinin-Cl. ml/min	Weight g
**Control**	At start	3.12 ± 2.40			105 ± 13.78
	Last	4.87 ± 3	3.23 ± 0.10	0.55 ± 0.80	140 ± 15.49

**Pre-Proteinuria**	At start > 3. inj.	4.59 ± 3.70			100 ± 15.80
		5.57 ± 2	3.02 ± 0.15	0.49 ± 0.80	

**Acute Nephrosis**	At start > 9.inj.	5.04 ± 2			101 ± 0.80
		91.34 ± 91*	2.50 ± 0.63**	0.38 ± 0.28**	

**Chronic Nephrosis**	At start > 7.inj.	5.18 ± 2.20			100 ± 8.94
		247.5 ± 90*	2.35 ± 0.38**	0.29 ± 0.10**	210 ± 24.49**

All data are presented as mean ± SD (median) and analyzed by Dunn Multiple Comparison Test following Kruskal Wallis ANOVA.

*p < 0.0025 **p < 0.05

For all groups their own control protein excretion (at start) is shown.

**Table 3:** Values of Proteinuria in acute nephrosis group/1-9. injections

Rats	Proteinuria (mg/24 hours)									
	
	**Before inj**.	**1.inj**.	**2.inj**.	**3.inj**.	**4.inj**.	5.inj.^a^	**6.inj**.	**7.inj**.	**8.inj**.	**9. inj**.
1^st^	5.30	5.80	5.50	6.55	11.37	17.42	15.24	19.82	27.74	47.52

2^nd^	3.39	4.62	6.43	8.22	11.32	14.35	18.52	17.68	24.22	41.55

3^rd^	7.18	6.31	6.72	9.96	16.32	15.68	12.69	17.36	30.20	131.90

4^th^	4.64	4.25	3.80	4.87	8.03	12.59	14.28	18.04	25.67	76.53

5^th^	4.58	4.20	4.67	6.46	6.41	12.56	19.67	26.95	33.16	67.91

6^th^	5.19	4.29	4.40	4.39	12.81	15.03	12.28	23.42	76.45	183.67


**Average**	**5.04**	**4.91**	**5.25**	**6.74**	**11.04**	**14.60**	**15.44**	**20.50**	**36.24**	**91.34**

**Standard dev**.	**± 2**	**± 1.4**	**± 1.4**	**± 3.2**	**± 5.2**	**± 2.8**	**± 4.2**	**± 6.4**	**± 40**	**± 91**

a. Proteinuria increased after the 5rd injection

### Light and electron microscopical analyses

After necropsy, the left kidney cortex was immediately divided into 1 mm3 pieces for transmission electron microscopy. They were firstly fixed in 4% glutaraldehyde (Sigma, G5882, USA) in a 0.1 M phosphate buffer solution (PBS), post-fixed by 1% OsO4 prepared in the same buffer solution, dehydrated with graded ethanol (Merck, Germany) and, embedded into araldite medium (G4901 Sigma Chemical Co St. Louis, MO, USA). Semi-thin sections were cut into 1 μm thickness by glass knives to help of the ultramicrotome (Reichert UM 2 and UM 3, Austria) and stained with 1% toluidine blue (prepared with 1% borax in bidistilled water). The sections were examined under a binocular light microscope (Leica, Germany) using immersion objective. Ultra-thin sections were obtained in 50 nm thickness onto copper grids (300 mesh) with the same microtome, stained with uranyl acetate and lead citrate and they were investigated by transmission electron microscope (Zeiss Electron Microscope 9 and Electron Microscope 10, Oberkochen, Germany).

### Immunohistochemical analyses

Cortical renal tissues were fixed in 10% neutral buffered formalin and embedded in paraffin. For detection of angiogenesis, TP/PD-ECGF (Thymidine Phosphorylase/Platelet Derived-Endothelial Cell Growth Factor) protein immunohistochemical staining was performed. The sections of 5 μm thickness were placed onto slides coated with poly-L-Lysine, (PLL, Sigma, St. Louis, MO) then deparaffinized in toluene and rehydrated in graded alcohol series. Histostain-Plus™ Broad Spectrum Kit (95-9943-B Zymed Lab. Ins. San Francisco CA, USA) was used for immunoperoxidase staining. Immunohistochemistry procedure was performed using a combination of microwave oven heating for antigen retrieval and standard streptavidin-biotin-peroxidase method. Endogenous peroxidase activity was blocked by hydrogen peroxide (3%). Each section was then incubated for 15 minutes at room temperature with blocking solution to block cellular peroxidase activity. Sections were incubated with anti-TP/PD-ECGF antibody (host range: human, mouse, rat; prediluted; LabVision Corp., USA) for 1 hour at room temperature, then washed with PBS. Specific staining was performed with biotinlated universal secondary antibody, horseradish peroxidase streptavidin-complex, and amino-ethyl-carbazole as chromogen. Sections were counter-stained with Mayer's hematoxylin. As for negative control, distilled water was performed instead of primary antibody and as positive control, a malignant melanoma tissue was used.

### Semi-quantitation of immunoperoxidase staining

PD-ECGF immunperoxidase reaction of the rats' glomeruli was evaluated by Hill's scoring system [33]. This evaluation has been done by two observers in all rats of each group in at least randomly chosen ten glomeruli in two different sections of same tissue and all values were summed in Table 4. Immunopositivity was scored from 0 to +4 (0 = none, +1 weak immunopositivity, +2 moderate immunopositivity, +3 strong immunopositivity and +4 very strong immunopositivity) (Table 4).

**Table 4:** In all groups, number of cases and immunostaining for PD-ECGF

Groups (n = 6)	Number of Cases	PD-ECGF
**Control**	6/6	-

**Pre-Proteinuria**	5/6	+

**Acute nephrosis**	4/6	++

**Chronic nephrosis**	5/6	++++

Immunpositivity is scored from 0 to 4 as (-none-, +1 weak immunpositivity, +2 moderate immunpositivity, + 3 strong immunpositivity, +4 very strong immunpositivity).

### Statistical Analysis of Total Angiogenic Stages

All stages of angiogenic fields are counted in 10 randomly selected glomerules of all groups in semi-thin sections (stained with toluidine blue) by two observers under light microscope (Olympus BX61) with 100x immersion objective. Averages of data are given in Table 5 as mean and standart deviation (median) and compared by One-way ANOVA followed by Dunn Multiple Comparison Test. In all comparisons, statistical significance was defined as *p *< 0.01.

**Table 5:** Total angiogenic stage counts in all groups

Groups (n = 6)	Average angiogenic stage counts in 10 glomerules
**Control**	0.12 ± 0.04

**Pre-Proteinuria**	1.08 ± 0.03**

**Acute nephrosis**	2.13 ± 0.14**

**Chronic nephrosis**	4.87 ± 0.21**

All data are presented as mean ± SD (median) and analyzed by Dunn Multiple Comparisons Test following One-way ANOVA.

**p < 0.01

### Statistical Analysis of Urine and Blood Data

Proteinuria, serum albumin, creatinine-clearance, weight data of all groups were given as mean ± standard deviation (median) and compared by Kruskal Wallis ANOVA followed by Dunn Multiple Comparison Test. In all comparisons, statistical significance was defined as *p *< 0.05 and *p *< 0.0025 (Table 4).

## Results

### Urine and blood analyses

In control and experimental groups, proteinuria (mg/24 hours), serum albumin (g/dl), creatinine clearance (ml/minute) and weight (g) are shown in Table 2. In all PAN induced rats, proteinuria increased suddenly and significantly after the fifth injection (6th day). The 'pre-proteinuria group' was just defined as the group two days before this rising of proteinuria (see Table 3). As it is shown in Table 2, proteinuria levels in control and pre-proteinuria group were not significantly different from each other (*p *< 0.0025). Their serum albumin, creatinine clearances and weights also were not significantly different (*p *< 0.05). However, proteinuria in the acute group significantly increased. Serum albumin and creatinine clearances significantly decreased.

In the chronic nephrosis group, proteinuria was remarkably higher than it was in the acute group and about significantly higher than it was in the controls (*p *< 0.0025). Serum albumin decreased significantly and creatinine clearance decreased to nearly half of that in the control group (*p *< 0.05) and it was accepted as an evidence of glomerulopathology. In the rats of the chronic group, body weight rise to the two-fold of that in the controls due to the increase in fluid resorption (*p *< 0.05) (Table 2).

### Immunohistochemistry

**Figure 1:**
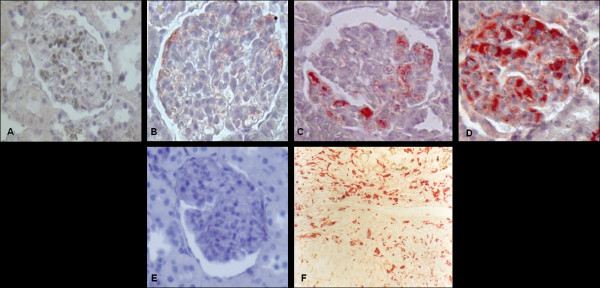
**Expression of PDECGF in the control and experimental groups**. Control group has no immunopositivity (A). Pre-proteinuria group has weak immunopositivity around the mesangial matrix (B). Acute group has moderate immunopositivity around the mesangial matrix regions (C). Chronic group has very strong immunopositivity in the cells of mesangial matrix regions (D). PDECGF- Negative chronic control group has no pozitivity (E) X40. Pozitive control is malignant melanoma tissue and has strong immunpozitivity in stromal cells (F) X20.

There was no immunreaction for PD-ECGF in the control group animals (Figure 1A) and negative control preparations (Figure 1E). In the pre-proteinuria group, a weak immunpositivity was detected (Figure 1B). The acute and chronic nephrosis groups showed a strong immunpositivity especially in the cells of mesangial matrix regions (Figure 1C, D; Table 4). Pozitive control (malignant melanoma) showed strong positivity (Figure F).

### Light microscopy

**Figure 2:**
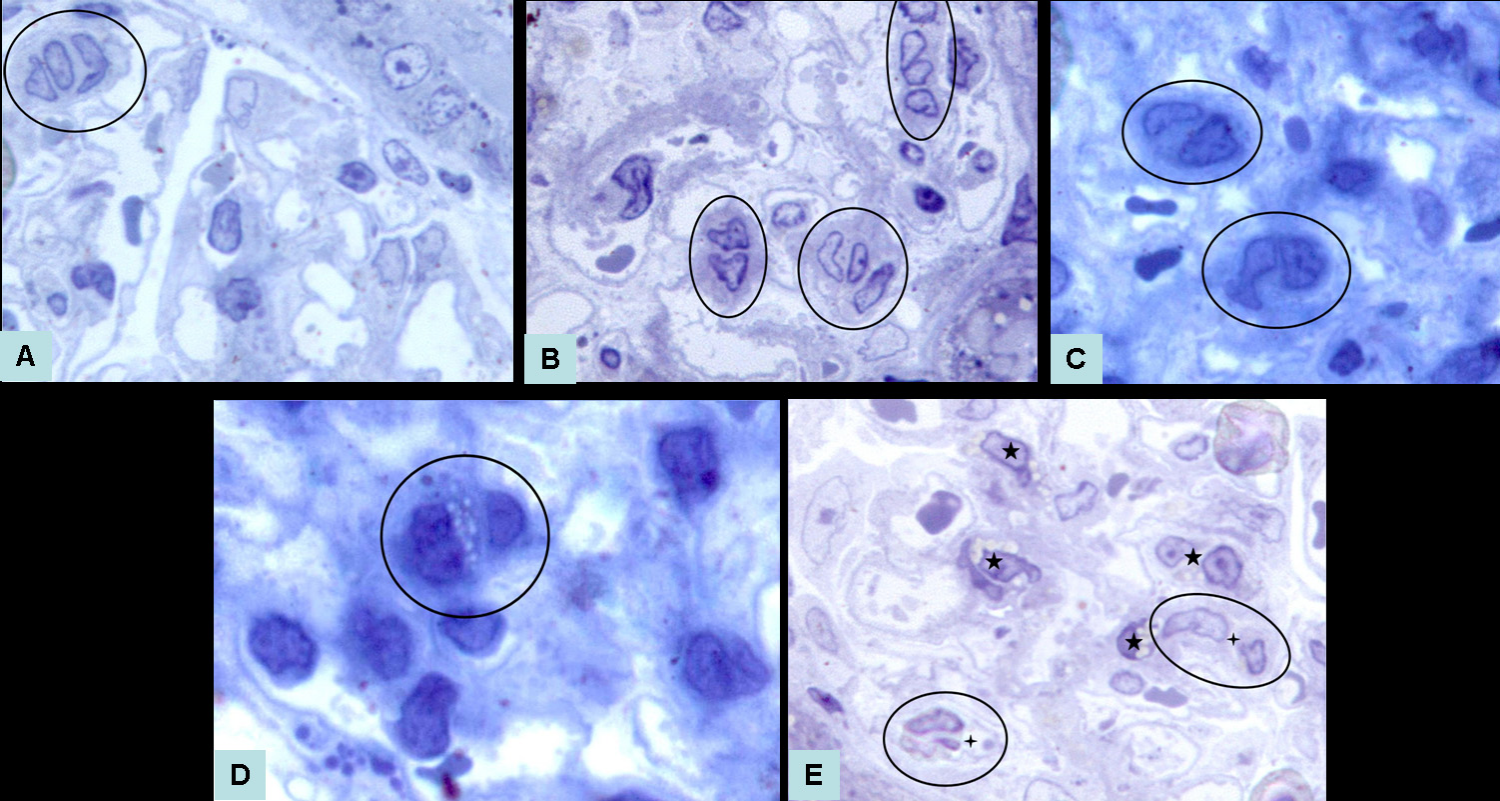
**(A) In an early stage of angiogenesis, an angiogenic cell island (circle) in the glomerulus without a basal lamina and lumen is seen in the semi-thin section of the acute group**. X100. (B) In an early stage of angiogenesis, angiogenic cell islands (circles) in the expanded mesangial matrix of the glomeruli are seen in the semi thin section of the chronic group. X100. (C) In the early polarization stage of angiogenesis, two immature capillaries (circles) located inside an expanded dense mesangial matrix of a glomerulus are seen in the semi thin section of the chronic group. Endothelial cells have dense basophilic cytoplasms. X100. (D) In lumen formation stage of angiogenesis, an immature capillary (circle) in an expanded dense mesangial matrix with several vacuoles inside the dense basophilic endothelial cytoplasm is shown in the semi thin section of chronic group glomeruli. X100. (E) In the semi thin section of the chronic group's glomeruli, many macrophages (five ended stars), and immature capillaries (circles) in advanced stage of lumen (four ended stars) formation are seen. X100.

In the acute and chronic group animals' glomeruli, dilation of existing capillaries with endothelial proliferation and expanded mesangial matrix were observed. Within the expanded mesangial matrix, loss of existing capillaries was generally observed while angiogenic cell groups containing bigger nuclei with peripherally located heterochromatin (Figure 2A, B) and immature capillaries which have endothelial cells with dense basophilic cytoplasm at the polarization stage (Figure 2C, D) were seen. In advanced lumen formation stage of polarization of immature capillaries, their endothelial cells were differentiated and their cytoplasm contained numerous vacuoles (Figure 2D). Immature capillaries were generally observed in adjacent macrophages. Some of these macrophages were 'foam-cell-like type macrophages' (Figure 2E).

When total angiogenic stages of semi-thin sections were counted under light microscope in 10 glomeruli of each rat in all groups, mean values showed that angiogenetic stage numbers of pre-proteinurea, acute and chronic nephrosis groups were significantly different from control group and showed gradually increasing manner in experimental groups (Table 5).

### Electron microscopy

**Figure 3:**
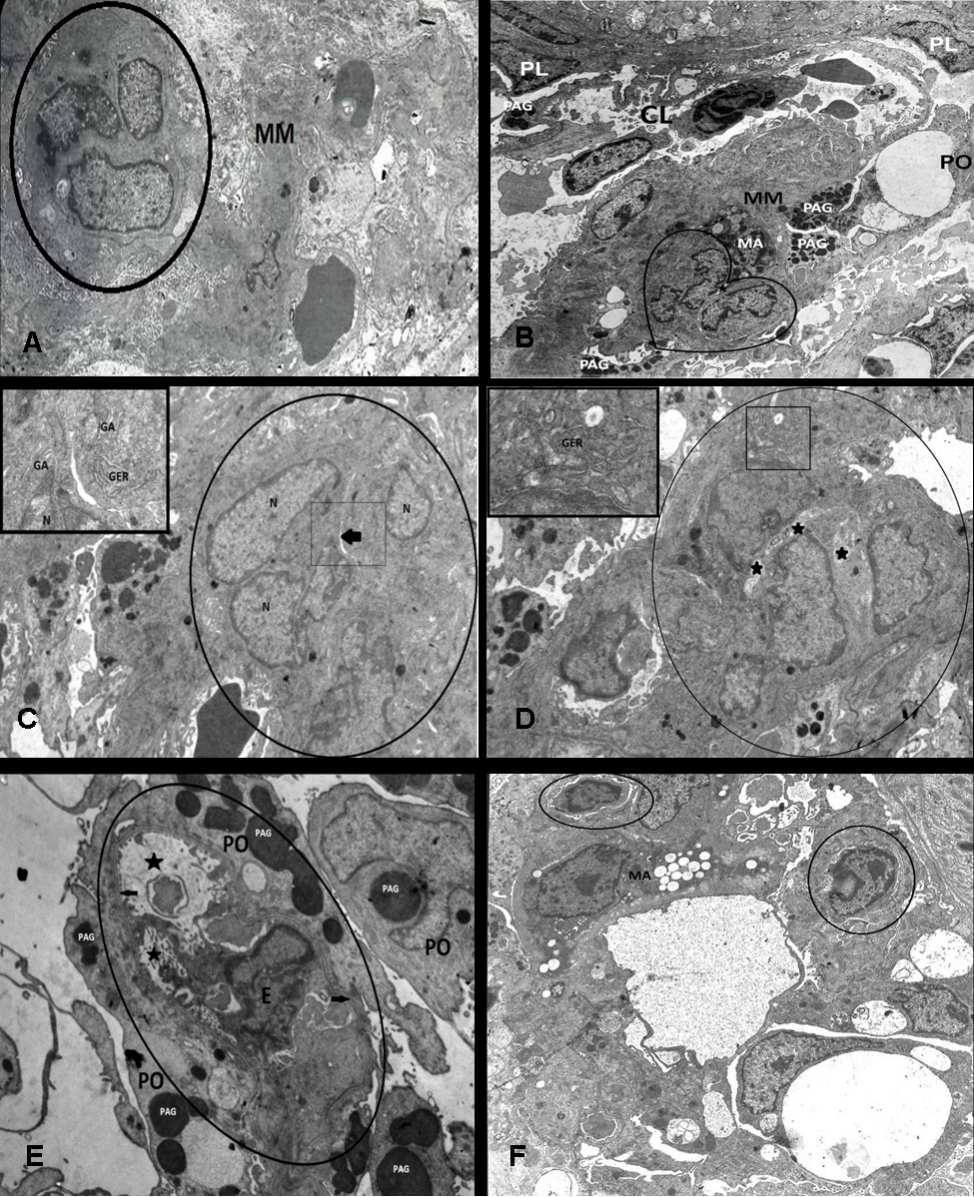
**(A) An electron micrograph from a glomerular area of the chronic group that shows an angiogenic cell island (circle) in enlarged mesangial matrix (MM) during early stage of angiogenesis**. Lumen and basal lamina are not seen yet at the cell island. X2000. (B) Immature capillary (hearth-shaped) with a primitive lumen (star) and a macrophage (MA) in an increased mesangial matrix (MM) adherent to existing capillaries (CL), PAG: protein absorption granules in podocyte (PO), PL: Parietal layer. x2470. (C) A glomeruli area of the acute group that shows an immature capillary (circle) in primitive lumen formation stage. A primitive lumen formation (arrow) is observed in the cell island. The enlarged granular endoplasmic reticulum (GER) cisterns and well developed Golgi apparatus (GA) are seen in the endothelial cell's cytoplasm (shown with high magnification in the inset at the upper left x25000). Around immature capillary, a basal lamina can not be seen yet. N: Nucleus. X5000. (D) An area from a glomerulus of the chronic group that shows an immature capillary (circle) in subsequent stage of primitive lumen (stars) formation.x7500 In their cytoplasm, developed granular endoplasmic reticulum cisterns (GER) are seen in the inset at the upper left. X25000. (E) An area from a glomerulus of the acute group that shows an immature capillary (circle) containing an advanced stage of the lumen (stars) with an apoptotic endothelial cell and cell debris. In the periphery of the immature capillary, the basement membrane (arrows) becomes clear. PO: Podocyte, E: Apoptotic Endothelial cell nucleus, PAG: Protein Absorption Granule × 7500. (F) An area from a glomerulus of the chronic group that shows two small immature capillaries at the lumen formation stage (circle) and a foam-cell like macrophage (Ma) nearby. X5400.

In the expanded mesangial matrix of the glomeruli in acute and chronic PAN, there was generally loss of existing capillaries but we showed the angiogenic cell islands without basal lamina and lumen (Figure 3A) and immature capillaries (Figure 3B-F). Angiogenic cells have big nuclei with peripherally located heterochromatin (Figure 3A). Furthermore, in some areas of acute and chronic PAN glomeruli, primitive lumen formation stages of polarization were observed in immature capillaries (Figure 3B-F). In these immature capillaries, endothelial cells were also differentiated (Figure 3B-F). We also observed basal lamina formation at the advanced polarization stage of immature capillaries (Figure 3E, F). In primitive lumen development stages of immature capillaries, endothelial cells had well developed GER cisterns and Golgi apparatus (Figure 3C, D). Moreover, in the advanced lumen formation stage of immature capillaries, we observed apoptotic endothelial cells (Figure 3E). There were macrophages in the close proximity of these immature capillaries. Some of these macrophages were 'foam-cell-like' type (Figure 3F).

## Discussion

Angiogenesis, the formation of new blood vessels from pre-existing vessels, is not only a critical step in embryogenesis and wound healing, but also contributes to the pathogenesis of tumor growth and chronic inflammation [8,10,21,23,24]. It is a complex process involving extensive interaction between the cells, soluble factors and extracellular matrix (ECM) components. Angiogenesis is initiated by vasodilatation and increased permeability. The formation of a vascular network requires different sequential steps including; the release of proteases from activated endothelial cells, degradation of the basement membrane surrounding the existing vessels, differentiation and migration of the endothelial cells into the interstitial space, endothelial cell proliferation forming tubular structures, lumen formation, generation of new basement membrane with the recruitment of pericytes and finally of fusion of newly formed vessels and initiation of blood flow [18,34-39].

As it is well known, hypoxia results in both angiogenesis and inflammation [27], and thus the production of growth factors increases [28-31]. Angiogenesis provides oxygen and nutrients for the metabolic needs of the cells that are present at inflammatory sites [18]. Therefore, we considered that the expanded avascular mesangial matrix regions of the glomeruli could induce the angiogenesis by creating a hypoxic state.

In our microscopic examinations, we observed an increased mesangial matrix with inflammatory areas including macrophages, and various steps of angiogenesis including vasodilatation and endothelial proliferation in pre-existing capillaries, as well as, angiogenic cell islands without basal lamina and lumen formation in immature capillaries containing basal lamina in glomeruli of acute and chronic PAN nephrotic rats. During angiogenesis, the transport of plasma proteins such as fibrinogen and plasminogen from the blood stream into the surrounding tissue is excessively increased. These plasma proteins provide a convenient environment for the migrating angiogenic cells [35]. In our light microscopic studies, we observed angiogenic cell islands and immature capillaries surrounded by dense mesangial matrix areas. We considered that this situation could be correlated with highly concentrated plasma proteins leaked out the pre-existing dilated capillaries around this area.

The angiogenic cell islands can be easily distinguished from the surrounding cells by their big nuclei with peripherally located heterochromatin. These cell islands had no basal lamina yet. When the lumen formation of angiogenic cell islands were completed after the polarization stage, the basal lumina has appeared [40]. As evidence to this argument, in our microscopic and ultrastructural images, we observed more basophilic cytoplasm of immature capillaries and prominent increase in the granular endoplasmic reticulum cisterns which is responsible for the formation of the basal lamina components. Moreover, some researchers have argued that endothelial apoptosis is effective on the primitive lumen formation stage of the angiogenic cell islands [40]. In our study, we observed apoptotic endothelial cells with advanced lumen formation of immature capillaries in our ultrastructural images as a support this argument.

In our microscopic and ultrastructural images, macrophages were the most abundant cell type in the angiogenic regions of the increased avascular matrix. Various cell types and cell products induce or modulate angiogenesis. The major of these cells are macrophages. In all steps of angiogenesis, macrophages take place by their secretary activity. It was reported that they had angiogenic characteristics when exposed to low oxygen supply [8]. Macrophages are among the main sources of metalloproteases (e.g. collagenases) and serine proteases (e.g. elastase and plasminogen activator). These enzymes can degrade ECM molecules, modulate the mechanical framework, and lead to the released ECM-bound growth factors. In addition to proteases, macrophages produce several factors that induce migration of endothelial cells. Most of them also support other stages of the angiogenic process such as proliferation or differentiation of endothelial cells. Their inducing effects on migration seem to be sufficient for the initial neovascularization as migrating endothelial cells can form sprouts without proliferation. Thus, macrophages release several factors that do not directly induce angiogenesis but act indirectly by attracting or activating angiogenic cells. This activity occurs in all phase of the angiogenic process [8,18,41]. Hence we considered that the macrophages in this increased avascular matrix were activated due to the presence of insufficient oxygen and induced the angiogenesis.

We also showed the existence of the foam cells in the expanded avascular mesangial matrix of glomeruli in PAN rats. In some diseases, e.g. focal and segmental glomerulosclerosis and hyalinosis, mesangial cells (MCs) exhibit foam cell like morphology. These lipid-laden MCs have impaired phagocytic capacity and disrupted cytoskeletons. Studies have demonstrated that IGF-1 (insulin-like growth factor-1) induces MC to transform into foam cell by phagocytosing lipids [42,43]. We considered that these cells frequently observed around the angiogenic regions could be also responsible from the development of angiogenesis like the other macrophages.

In scarred kidneys, it was reported that PD-ECGF expression is elevated and its level of expression is correlated with pathological angiogenesis [17,20,22-24]. In this study, we detected occasional angiogenic regions in expanded avascular mesangial matrix of the glomeruli in acute and chronic PAN induced rats. We showed elevated PD-ECGF immunostaining in the glomeruli of acute and chronic PAN induced rats. When we compared control and experimental groups, number of total angiogenic stages in experimental groups increased in parallel with PD-ECGF immuno-reactivity results (Table 4, 5). These results supported our ultrastructural findings observation related to angiogenesis development. Thus these findings are consistent with the results of recent studies that have indicated that PD-ECGF expression level is correlated with the number of microvessels in various pathological conditions and may contribute to neovascularization [17,18,20,22-24]. Therefore, as many researchers, we expect that PD-ECGF may become one of the major targets of angiogenesis therapy in future [44].

## Conclusions

In this study, we have thought that loss of existing capillaries within the increased mesangial matrix might cause hypoxia and thereby induce angiogenesis in acute and chronic PAN. Our findings have suggested that acute and chronic PAN causes progressively increased PD-ECGF expression and induce the angiogenesis in the glomeruli.

## Abbreviations

bFGF: basic-fibroblast growth factor; ECM: extracellular matrix; HGF: hepatocyte growth factor; IGF-1: insulin-like growth factor-1; MCs: mesangial cells; PAG: protein absorbtion granules; PA: puromycine aminonucleoside; PAN: puromycine aminonucleoside nephrosis; PBS: phosphate buffer solution; PD-ECGF: protein platelet-derived endothelial cell growth factor; TCA: modified trichlor acetyl acid method; VEGF: vascular endothelial growth factor.

## Competing interests

The authors declare that they have no competing interests.

## Authors' contributions

Ismail Seckin, Sibel Kokturk and Mumin Uzunalan designed the experimental procedure and carried out the immunohistochemistry; Huseyin Sonmez and Zeynep Öztürk performed biochemical studies and evaluate the biochemical results; Meltem Pekpak performed clinical evaluations; light and electron microscopic studies were performed and evaluated by Ismail Seckin, Mumin Uzunalan, Sibel Demirci and Elif Yaprak; Ismail Seckin and Elif Yaprak performed the statistical analysis; Ismail Seckin, Meltem Pekpak, Sibel Demirci and Elif Yaprak drafted the manuscript. All authors read and approved the final manuscript.
